# Self-reported head trauma among Native Americans who inject methamphetamine: a cross-sectional study

**DOI:** 10.3389/fpubh.2025.1588332

**Published:** 2025-08-13

**Authors:** Monica R. Lininger, Michael P. Anastario, Aaron Specht, Paula Firemoon

**Affiliations:** ^1^Center for Health Equity Research, Northern Arizona University, Flagstaff, AZ, United States; ^2^Department of Physical Therapy and Athletic Training, Northern Arizona University, Flagstaff, AZ, United States; ^3^Department of Health Sciences, Northern Arizona University, Flagstaff, AZ, United States; ^4^School of Health Sciences Purdue University, West Lafayette, IN, United States; ^5^Fort Peck Community College, Poplar, MT, United States

**Keywords:** methamphetamine, traumatic brain injury, head trauma, injection drug use, Native Americans

## Abstract

**Introduction:**

Traumatic brain injury (TBI) is a significant public health concern, with disparities in prevalence and care access among Native Americans. The syndemic relationship between substance use and TBI remains underexplored in Native Americans who inject methamphetamine, a population at high risk for both conditions. This study examines the association between self-reported TBI and substance use patterns in a sample of Native Americans who inject methamphetamine.

**Methods:**

In this cross-sectional study, 60 Fort Peck Tribal members who reported injecting methamphetamine were recruited. Data collection included anthropometric measures, a structured questionnaire (lifetime TBI history, health conditions, and substance use characteristics), and portable X-ray fluorescence to measure tibial lead (Pb) concentrations. Logistic regression analyzes assessed associations between self-reported TBIs and substance use patterns stratified by gender.

**Results:**

Self-reported lifetime TBI prevalence was 42%. Among females, cumulative years of sedative (OR: 1.3, 95% CI: 1.0–1.5) and cocaine use (OR: 1.3, 95% CI: 1.0–1.5) were associated with increased TBI reports. For males, hypertension (OR: 754.6, 95% CI: 10.7–53,294.1) was a significant predictor. Elevated tibial Pb levels were associated with increased TBI risk in both females and males.

**Discussion:**

Findings highlight the syndemic burden of substance use and TBI in Native Americans who inject methamphetamine. Gender-specific risk factors suggest targeted interventions are needed. The study underscores the need for increased representation of Native Americans in concussion research and supports implementing TBI screening within substance use treatment programs.

## 1 Introduction

Traumatic brain injury (TBI) represents a significant public health concern in the United States (US), with estimates of approximately 2.8 million TBI-related emergency department visits, hospitalizations, and deaths annually (1). These estimates have increased by 50% in nearly the past two decades (1) and may be under-reported as not all individuals seek medical care following a head injury (2–5). The prevalence of self-reported lifetime concussion among adults has been documented to be between 21.7% (6) and as high as 28.9% (7). Even before the drastic rise in prevalence, TBIs were referred to as a “silent epidemic.” Disparities exist across populations, with rural residents facing unique challenges in accessing appropriate care for concussions and mild TBIs (i.e., concussions), potentially leading to underreporting and inadequate treatment (8). Specifically, American Indians and Alaskan Natives (AI/ANs), when compared to other racial and ethnic groups, consistently have higher rates of TBIs (9–11).

Substance use may contribute to excess disease burdens associated with TBI. Substance use disorder (SUD) encounters in emergency departments accounted for over $13 billion in 2017 (9). The intersection of TBI and SUDs has been characterized by a complex bidirectional relationship (12, 13). Individuals with a history of TBI exhibit a heightened vulnerability to SUDs due to neurophysiological changes that impair executive functioning and foster risk-taking behaviors such as substance misuse (13, 14). Concurrently, substance and intoxication significantly increase the risk of sustaining a TBI (13, 15–18). This bidirectional relationship exacerbates adverse health outcomes such as impaired recovery from head trauma (19), increased risk for future injury (13) and mood disorders (20). When speaking of substance use, more literature focuses on alcohol consumption (14, 21–24) and not necessarily other drug use.

Since 2000, there has been a dramatic increase in the use of methamphetamine by individuals 12 years of age or older (25–27), with the highest rates among those 35–39 (27). Furthermore, in 2017, the greatest use of methamphetamines was by non-Hispanic AI/ANs (4.3%) (27). With the rise in the use of methamphetamine, there has been a 30-fold increase in the associated mortality rate (25, 26), and Native Americans have the fastest and highest rates (28–30).

Syndemics describe the ways that disease interactions and concentrations exist in socially marginalized populations where disease processes cannot be reduced to a single causal variable at the individual level (31–34). Substance use and TBI may be concentrated in historically marginalized communities, including Native Americans, who face disproportionate exposure to systemic barriers, high rates of substance use, and increased vulnerability to TBIs. Understanding the syndemics (i.e., clustering of substance use and head trauma) facing Native Americans who inject drugs is critical. Due to the sparse literature on this association and Indigenous people who inject drugs generally, the purpose of this work is to determine the association between self-reported traumatic head injuries and substance use patterns in a sample of Native Americans who inject methamphetamine.

## 2 Methods

### 2.1 Participants

This work is part of a larger community-engaged project (35). Briefly, the research team has conducted community-engaged research with the Fort Peck Tribes for the past 7 years. This specific project used a cross-sectional design with 60 Fort Peck Tribal members who all reported current use of injecting methamphetamine. The Fort Peck Indian Reservation has approximately 8,000 tribal members within the 2.1 million acre reservation in Northeastern Montana (36).

For this cross-sectional survey-based study, a chain referral sampling method (37–39) was implemented as the population is highly connected (40, 41), and word-of-mouth strategies have succeeded in previous research with this population. Inclusion criteria included being a registered or associated member of a federally recognized tribe, ≥18 years of age, and participants had to have injected methamphetamine at least once in the month preceding data collection. The only exclusion criterion was current incarnation or in police custody at the time of data collection. Following verbal consent, eligible participants were enrolled in the Institutional Review Board (IRB [Fort Peck Tribal IRB on November 15, 2023 and Northern Arizona University IRB (2133585-2)] approved project). Participants received a $50 gift card along with the option to take home fentanyl test strips, sterile syringes, and naloxone after the study.

### 2.2 Measures and procedures

Following the consenting process, anthropometric measures of height and weight were taken. Next, a questionnaire was verbally administered, face-to-face, by a trained interviewer at the doctoral level (PhD or MD). Questionnaires were used to collect demographic information and assess substance use history, sources of potential metal exposures, and injection preparation filtration practices. Finally, portable x-ray fluorescence (pXRF) was used to evaluate metal concentrations in the tibial bone of the participant.

### 2.3 Instrumentation

#### 2.3.1 Demographic information

The questionnaire included self-reported gender identity, age, year of birth, relationship status, tribal registration or associate tribal member status, and tribal identification (Assiniboine, Sioux, both, other). Anthropometric measures of weight and height were collected using a digital scale and a standard tape measure securely affixed to a wall, respectively. Height and weight were then used to calculate body mass index (BMI) following standardized procedures (42).

#### 2.3.2 Health conditions

Participants were asked 13 items specific to their self-reported health conditions with the prompt of “Has a health professional such as a doctor, nurse, nurse practitioner, or a community health worker ever told you that you have one of the following? If so, have you received treatment?” Conditions included Hepatitis C, HIV, opioid use disorder, mental health diagnosis, diabetes, hypertension, heart and liver disease, and cancer. Binary response options (yes/no) were provided for every diagnosis, received treatment, currently have a health condition, actively receiving treatment.

#### 2.3.3 Substance use

Cumulative years of substance use for various substances were assessed using a version of the Substance Use Inventory adapted from the Addiction Severity Index 5th Edition adapted for use with Native Americans (38, 43). This module evaluates participants’ usage of substances such as alcohol, heroin, methadone, other opiates/analgesics, barbiturates, sedatives/hypnotics/tranquilizers, cocaine, amphetamines, cannabis, hallucinogens, and inhalants. For each substance, participants were asked about lifetime use (yes/no), use within the past 30 days (yes/no), age of first use, total years of use, route of administration, and age at last use.

#### 2.3.4 Self-reported traumatic head injury

Self-reported lifetime history of mTBI and concussion is now commonly seen in literature to better estimate traumatic head injuries due to the significant underreporting of seeking medical care (7, 44, 45). Participants were asked two questions related to head trauma: one for lifetime incidence and seeking medical care and then not seeking medical care. More specifically, the two questions were “Have you ever hit your head and had a doctor or other health professional tell you that you had a concussion?” and “Have you ever had a concussion and not seen a doctor or other health professional?” For each item, response options were no, once, 2–4 times, 5 times or more.

#### 2.3.5 Lead concentration in tibial bone

Tibial bone Pb concentration was measured using a Thermo Niton Xl3t GOLDD+ portable XRF analyzer. Bone metals can reflect more than 90% of body burden of heavy metal exposure and can be used to reflect up to three decades of past exposure to Pb (46, 47), and was both hypothesized and measured due to the potential for the consumption of Pb contaminated methamphetamine over the lifespan. The mid-tibia was cleaned with an alcohol swab, and participants rested in a chair with the leg being measured extended parallel to the ground and the foot resting on an adjacent chair. The operator identified the mid-tibia and verified placement by feeling the tibia below and on either side of the device. Counts from elemental peaks were converted to ug/g dry bone from the calibration phantom measurements and ultimately converted to ug/g bone mineral using a conversion factor from dry bone to bone mineral.

### 2.4 Statistical analysis

Using STATA (Release 18, College Station, TX: StataCorp LLC), descriptive statistics were calculated for continuous (e.g., age, BMI, years of substance use, age of first injection) using means and standard deviations. For categorical variables (e.g., gender, self-reported health conditions, etc.), frequencies and percentages were calculated. Next, bivariate tests were analyzed with self-reported concussion and seeking medical care as the outcome variable (never, 1 time, 2–4 times, and ≥ 5 times). Ordinal logistic regression was used to calculate the odds ratios (OR), adjusted odds ratios (aOR), and 95% confidence intervals (95% CI) for independent variables of interest while controlling for potential confounders. Model fit was assessed using pseudo *R*^2^. All bivariate tests and regression models were stratified by self-reported gender identity (male and female) due to known differences in self-reported concussions (7, 48, 49).

## 3 Results

Of the 60 participants, there was an even split of males and females (Table 1), approximately 42 years of age, and nearly all were registered members of a federally recognized tribe (96.7%). On average, the longest use of specific substances included alcohol and cannabis, both self-reported for 24 years. Methamphetamine was the second longest used substance for an average of 19 years. The most prevalent health condition was Hepatitis C (43.3%), with the least frequent being HIV (1.8%) and heart disease (3.5%). Forty-two percent (25/60) of the sample reported at least one previous TBI across their lifetime, with males having a slightly higher percentage (13/30 = 43%) than females (12/30 = 40%).

**Table 1:** Sample characteristics and self-reported concussion among Indigenous people who inject methamphetamine, *n* = 60.

Demographic variables		Percent (%) or Mean (SD)
Gender* (% female)	50.0%
Age (mean, SD)	42.0 (9.9)
Registered member of a federally recognized tribe (%)	96.7%
Associate Tribal member	3.3%
BMI (mean, SD)	26.5 (5.4)
*Substance use*	
Cumulative years used	
Alcohol (mean, SD)	24.1 (10.6)
Heroin (mean, SD)	1.5 (5.7)
Methadone (mean, SD)	0.2 (1.5)
Other opiates/analgesics (mean, SD)	5.6 (8.1)
Sedatives/hypnotics/tranquilizers (mean, SD)	1.2 (4.8)
Cocaine (mean, SD)	3.6 (7.9)
Methamphetamine/amphetamine (mean, SD)	19.4 (9.0)
Cannabis (mean, SD)	24.3 (14.0)
Hallucinogens (mean, SD)	1.0 (4.3)
Inhalants (mean, SD)	1.5 (4.4)
Overall # substances used, lifetime (mean, SD)	5.6 (1.9)
Overall # substances currently using (mean, SD)	2.7 (1.0)
Health conditions	
Hep C (%)	43.3%
HIV (%)	1.8%
Other infectious disease (%)	12.3%
Opioid use disorder (%)	15.8%
Other addiction or dependency diagnosis (%)	35.1%
Mental health diagnosis (%)	26.3%
Diabetes (%)	10.5%
Hypertension (%)	14.0%
Heart disease (%)	3.5%
Liver disease (%)	8.8%
Cancer (%)	8.8%
Abscesses (%)	7.0%
Other (%)	10.5%
Pb concentration in tibia bone (mean, SD)	12.1 (4.7)
Self-reported concussion (%)	41.7%

* Respondents only identified as male and female. %, percent; SD, standard deviation; #, number; Hep C, Hepatitis C Virus; HIV, Human Immunodeficiency Virus; Pb, lead.

For women, there was a statistically significant association between cumulative years of sedative and cocaine use and the number of self-reported head traumatic events where they sought medical care (Table 2). Those with a longer history of sedative (OR: 1.3; 95% CI: 1.0, 1.5) and cocaine use (OR: 1.3, 95% CI: 1.0, 1.5) had more self-reported concussions. When controlling for age, hypertension, years of methamphetamine and alcohol use, the overall number of substances, regardless of type, used across a lifetime (OR: 1.7; 95% CI: 1.1, 2.7) and tibial Pb concentration (OR: 1.2; 95% CI: 1.0, 1.5) were both significantly associated with the number of self-reported traumatic head events in this sample of women, showing that with longer use of substances and increase in tibial Pb concentration the likelihood of self-reported concussion increases (Table 3).

**Table 2:** Relationship between TBI and variables of interest in females only.

	Ever hit head and had doctor or other health professional tell you that you had a concussion				OR	95% CI	*p*-value
	Never	1 time	2–4 times	≥5 times			
	(*n* = 18)	(*n* = 4)	(*n* = 7)	(*n* = 1)			
Demographic variables							
Age (mean, SD)	41.6 (9.0)	46.5 (11.3)	42.1 (7.0)	35.0 (.)	1.0	(0.9, 1.1)	0.898
Registered member of a federally recognized tribe (%)	94.4%	100.0%	100.0%	100.0%	5315533.0	(0.0, ∞)	0.307
Associate Tribal member (%)	5.6%	0.0%	0.0%	0.0%	0.0	(0.0, ∞)	0.995
BMI (mean, SD)	26.4 (4.6)	30.9 (8.0)	27.4 (3.9)	23.8 (.)	1.0	(0.9, 1.2)	0.567
*Substance use characteristics*							
Cumulative years used							
Alcohol (mean, SD)	22.9 (9.2)	21.5 (9.1)	25.9 (7.3)	22.0 (.)	1.0	(0.9, 1.1)	0.621
Heroin (mean, SD)	0.0 (0.0)	0.0 (0.0)	0.0 (0.0)	27.0 (.)	1.0	(omitted)	NA
Methadone (mean, SD)	0.0 (0.0)	0.0 (0.0)	1.7 (4.5)	0.0 (.)	1.2	(0.9, 1.6)	0.185
Other opiates/analgesics (mean, SD)	5.2 (7.4)	12.3 (11.1)	7.0 (10.0)	10.0 (.)	1.0	(1.0, 1.1)	0.354
Sedatives/hypnotics/tranquilizers (mean, SD)	0.0 (0.0)	1.8 (3.5)	3.3 (7.8)	13.0 (.)	1.3	(1.0, 1.5)	0.009*
Cocaine (mean, SD)	1.1 (3.6)	0.3 (0.5)	3.7 (5.4)	16.0 (.)	1.3	(1.0, 1.5)	0.016*
Methamphetamine/amphetamine (mean, SD)	19.5 (8.6)	19.3 (4.3)	23.7 (6.0)	19.0 (.)	1.1	(1.0, 1.2)	0.319
Cannabis (mean, SD)	22.3 (12.4)	28.3 (14.5)	28.3 (8.0)	24.0 (.)	1.0	(1.0, 1.1)	0.241
Hallucinogens (mean, SD)	1.6 (5.0)	0.0 (0.0)	0.0 (0.0)	0.0 (.)	0.2	(0.0, ∞)	0.994
Inhalants (mean, SD)	2.1 (5.0)	0.0 (0.0)	4.1 (9.0)	0.0 (.)	1.0	(0.9, 1.2)	0.721
Overall # substances used, lifetime (mean, SD)	5.1 (1.8)	6.0 (2.0)	6.6 (2.5)	8.0 (.)	1.5	(1.0, 2.1)	0.037*
Overall # substances currently using (mean, SD)	2.6 (0.8)	2.5 (1.3)	2.9 (1.5)	5.0 (.)	1.5	(0.8, 2.8)	0.254
Health conditions							
Hep C (%)	44.4%	50.0%	71.4%	0.0%	1.7	(0.4, 7.0)	0.484
HIV (%)	0.0%	0.0%	0.0%	0.0%	1.0	(omitted)	NA
Other infectious disease (%)	0.0%	25.0%	16.7%	0.0%	4.9	(0.5, 52.7)	0.186
Opioid use disorder (%)	23.5%	25.0%	33.3%	0.0%	1.2	(0.2, 6.2)	0.855
Other addiction or dependency diagnosis (%)	23.5%	75.0%	50.0%	0.0%	2.6	(0.6, 11.7)	0.211
Mental health diagnosis (%)	23.5%	50.0%	33.3%	0.0%	1.4	(0.3, 6.8)	0.647
Diabetes (%)	17.6%	0.0%	16.7%	0.0%	0.5	(0.0, 5.9)	0.597
Hypertension (%)	17.6%	0.0%	0.0%	100.0%	0.7	(0.1, 8.7)	0.791
Heart disease (%)	0.0%	0.0%	0.0%	0.0%	1.0	(omitted)	NA
Liver disease (%)	0.0%	25.0%	16.7%	0.0%	4.9	(0.5, 52.7)	0.186
Cancer (%)	11.8%	25.0%	0.0%	0.0%	0.6	(0.0, 6.2)	0.621
Abscesses (%)	11.8%	25.0%	16.7%	0.0%	1.3	(0.2, 9.5)	0.771
Other (%)	11.8%	0.0%	16.7%	100.0%	3.4	(0.4, 33.6)	0.289
Pb concentration in tibia bone (mean, SD)	12.7 (4.5)	14.5 (5.4)	13.1 (6.3)	26.9 (.)	1.1	(1.0, 1.3)	0.198

*Statistically significant at 0.05. %, percent; SD, standard deviation; OR, odds ratio, 95% CI, 95% confidence interval; #, number; Hep C, Hepatitis C Virus; HIV, Human Immunodeficiency Virus; Pb, lead.

**Table 3:** Ordinal regression model for variables of interest and TBI.

	Females			Males		
	*a*OR (SE)	95% CI	*P* value	OR (SE)	95% CI	*P* value
Age	1.0 (0.6)	(0.9, 1.2)	0.451	0.9 (0.7)	(0.8, 1.1)	0.477
Overall # substances used, lifetime	1.7 (0.4)	(1.1, 2.7)	0.018*	0.8 (0.3)	(0.4, 1.6)	0.467
Hypertension	0.1 (0.2)	(0.003, 4.3)	0.236	754.6 (1639.1)	(10.7, 53294.1)	0.002*
Cumulative years of alcohol use	1.1 (0.09)	(0.97, 1.3)	0.126	0.9 (0.07)	(0.8, 1.0)	0.130
Cumulative years of methamphetamine/amphetamine use	1.02 (0.06)	(0.92, 1.3)	0.731	1.04 (0.06)	(0.9, 1.2)	0.421
Pb concentration in tibia bone	1.2 (0.1)	(1.0, 1.5)	0.047*	1.9 (0.4)	(1.1, 3.0)	0.005*

Model fit: x^2^_6_ = 12.31, *p* = 0.0554, Pseudo *R*^2^ = 0.21 (females); x^2^_6_ = 22.08, *p* = 0.001, Pseudo *R*^2^ = 0.37 (males). aOR, adjusted odds ratio; SE, standard error; 95% CI, 95% confidence interval; #, number. *statistically significant at 0.05.

The only statistically significant association for males was between self-reported hypertension and the number of self-reported head traumatic events where they sought medical care (Table 4). Those with hypertension had more self-reported concussions (OR: 39.7; 95% CI: 2.4, 651.6). When controlling for age, the number of substances used across a lifespan, and years of methamphetamine and alcohol use, hypertension (OR: 754.6; 95% CI: 10.7, 53294.1), and tibial Pb concentration (OR: 1.9; 95% CI: 1.2, 3.0) were both significantly associated with the number of self-reported traumatic head events (Table 3).

**Table 4:** Relationship between TBI and variables of interest in males only.

	Ever hit head and had doctor or other health professional tell you that you had a concussion				OR	95% CI	*P*-value
	Never	1 time	2–4 times	≥5 times			
	(*n* = 17)	(*n* = 9)	(*n* = 2)	(*n* = 2)			
Demographic variables							
Age (mean, SD)	41.7 (11.5)	41.8 (12.9)	41.5 (0.7)	44.5 (12.0)	1.0	(0.9, 1.1)	0.871
Registered member of a federally recognized tribe (%)	94.1%	100.0%	100.0%	100.0%	3422263.0	(0.0, ∞)	0.281
Associate Tribal member (%)	5.9%	0.0%	0.0%	0.0%	0.0	(0.0, ∞)	0.994
BMI (mean, SD)	25.5 (4.5)	26.1 (8.6)	29.8 (6.1)	24.1 (2.4)	1.0	(0.9, 1.1)	0.711
*Substance use characteristics*							
Cumulative years used							
Alcohol (mean, SD)	24.8 (11.2)	21.8 (16.1)	28.5 (2.1)	35.5 (7.8)	1.0	(1.0, 1.1)	0.698
Heroin (mean, SD)	1.3 (5.3)	3.3 (9.3)	0.0 (0.0)	4.0 (5.7)	1.0	(0.9, 1.1)	0.576
Methadone (mean, SD)	0.0 (0.0)	0.0 (0.0)	0.0 (0.0)	0.0 (0.0)	1.0	(omitted)	NA
Other opiates/analgesics (mean, SD)	4.8 (8.4)	2.3 (5.2)	6.0 (8.5)	9.5 (13.4)	1.0	(0.9, 1.1)	0.964
Sedatives/hypnotics/tranquilizers (mean, SD)	1.6 (6.8)	0.2 (0.7)	0.0 (0.0)	0.0 (0.0)	0.9	(0.6, 1.3)	0.561
Cocaine (mean, SD)	6.8 (11.4)	1.6 (4.0)	13.0 (18.4)	0.0 (0.0)	1.0	(0.9, 1.0)	0.367
Methamphetamine/amphetamine (mean, SD)	18.5 (11.0)	19.8 (10.6)	21.0 (1.4)	7.0 (1.4)	1.0	(0.9, 1.1)	0.668
Cannabis (mean, SD)	24.7 (15.6)	22.9 (19.1)	28.0 (0.0)	20.5 (29.0)	1.0	(1.0, 1.0)	0.832
Hallucinogens (mean, SD)	1.8 (6.3)	0.0 (0.0)	0.0 (0.0)	0.0 (0.0)	0.1	(0.0, ∞)	0.992
Inhalants (mean, SD)	1.2 (2.7)	0.0 (0.0)	0.0 (0.0)	1.5 (2.1)	0.8	(0.5, 1.3)	0.338
Overall # substances used, lifetime (mean, SD)	5.7 (1.9)	5.0 (1.1)	4.5 (0.7)	8.0 (1.4)	1.0	(0.6, 1.5)	0.928
Overall # substances currently using (mean, SD)	2.6 (0.9)	2.4 (1.2)	3.0 (0.0)	3.5 (0.7)	1.2	(0.6, 2.4)	0.632
Health conditions							
Hep C (%)	29.4%	44.4%	100.0%	0.0%	1.9	(0.4, 7.8)	0.394
HIV (%)	5.9%	0.0%	0.0%	0.0%	0.0	(0.0, ∞)	0.996
Other infectious disease (%)	11.8%	25.0%	50.0%	0.0%	2.1	(0.4, 12.0)	0.422
Opioid use disorder (%)	5.9%	12.5%	0.0%	0.0%	1.1	(0.1, 14.5)	0.963
Other addiction or dependency diagnosis (%)	41.2%	25.0%	0.0%	50.0%	0.5	(0.1, 2.5)	0.409
Mental health diagnosis (%)	23.5%	12.5%	50.0%	50.0%	1.5	(0.3, 8.3)	0.644
Diabetes (%)	5.9%	0.0%	50.0%	0.0%	2.3	(0.1, 37.7)	0.556
Hypertension (%)	5.9%	0.0%	50.0%	100.0%	39.7	(2.4, 651.6)	0.010*
Heart disease (%)	11.8%	0.0%	0.0%	0.0%	0.0	(0.0, ∞)	0.992
Liver disease (%)	17.6%	0.0%	0.0%	0.0%	0.0	(0.0, ∞)	0.993
Cancer (%)	5.9%	0.0%	50.0%	0.0%	2.3	(0.1, 37.7)	0.556
Abscesses (%)	0.0%	0.0%	0.0%	0.0%	1.0	(omitted)	NA
Other (%)	11.8%	0.0%	0.0%	0.0%	0.0	(0.0, ∞)	0.992
Pb concentration in tibia bone (mean, SD)	9.7 (3.2)	12.2 (3.2)	12.8 (6.7)	10.2 (0.2)	1.2	(1.0, 1.5)	0.112

*Statistically significant at 0.05. %, percent; SD, standard deviation; OR, odds ratio, 95% CI, 95% confidence interval; #, number; Hep C, Hepatitis C Virus; HIV, Human Immunodeficiency Virus; Pb, lead.

## 4 Discussion

In this work, we evaluated the association between substance use and head trauma in a cohort of Native Americans who inject methamphetamine. This work directly addresses a syndemics in a marginalized population high rates of polysubstance use and increased risk of TBIs, especially for the females in the present sample. While the exact reasons that females experience more concussions than males (50–52) remain unknown in this sample, a differentiating factor was length and type of drug use. Females with a more extended history of sedative and cocaine use had a higher number of self-reported concussions across their lifetime. Veliz et al. (45) found that females were more likely to have an association between nonmedical prescription drug use and the presence of concussion compared to males, although the sample was adolescents. One hypothesis is that those with an established pattern of drug use display more risky behaviors. Sensation-seeking or risky behaviors have been defined as personality traits involving the desire for intense experiences and sensations and a willingness to participate in physical and social risks (53, 54).

Interestingly, these results were not similar for males. Significant results centered more on hypertension, with an increase in the number of traumatic head events. In a recent study, Ozono et al. (55) found that individuals approximately 55 years of age with hypertension had more significant cognitive impairments compared to those without the modifiable risk factor following a TBI. The risk of hypertension following TBI was assessed in a prospective longitudinal cohort study with a 10 year follow up period (56). Izzy et al. (56) postulated that a TBI of any severity was significantly associated with an increased risk of hypertension when compared to those without a TBI in the matched (age, sex, race, and frequency of TBI) study. Although the present results are similar to Izzy et al. (56), their sample was starkly different, including primarily White (77%) participants with only 4% self-selected as Other (*n* = 157), which included Asian, Asian Pacific Islander, Native Hawaiian, American Indian, and Middle Eastern, showing the continued underrepresentation of Native Americans in published work, specific to the connection of head trauma and substance use.

Striking was the significance of Pb concentration levels with self-reported TBI for both males and females in the sample. Bone metal concentrations of Pb can reflect up to three decades of past exposure, and generally increase with age (47). Case studies have identified Pb toxicity among people who inject methamphetamine as a potential health concern due to the potential contamination of illicit batches created in clandestine laboratories (57, 58), but little systematic research on Pb bioaccumulation or exposure appears in the extant literature for people who inject methamphetamine. It is well established that Pb is neurotoxic and that there is no safe level of exposure to Pb (59). While our findings are cross-sectional, it is reasonable to assume that the neurotoxic effects of chronic lead exposures over the lifespan may increase the likelihood of TBIs, but the direction of the effect is likely more complex when considering the human lifespan and analysis of this relationship in a longitudinal study would further elucidate the nature of this association.

Because of the likelihood of non-reporting following a concussion, population estimates can be inconsistent. Self-reported lifetime concussion among adults has been reported between 21.7% (6) and 28.9% (7). However, for AI/ANs, while the precise percentage varies depending on the source, the fact remains that their prevalence is higher. In this small sample of 60 Native Americans, the lifetime self-reported concussion was nearly 42%. This sample could be an anomaly or may support the lack of representation of AI/ANs in the literature, specific to racial classifications for analytic procedures (60, 61). However, given the low engagement of AI/ANs who use injection drugs with medical care and treatment, targeted TBI screens for health encounters with AI/AN people who inject methamphetamine may be warranted.

### 4.1 Limitations

This work is novel and certainly impactful due to the lack of representation of AI/ANs in the literature, there are noted limitations. The most significant is the small sample size of sixty participants. However, the value of this pilot work with a notoriously difficult to access population provides a clear direction for research at a larger scale; therefore, the benefits outweigh the small sample size. The cross-sectional nature of the work limits the ability to contribute to the question of directionality (12) regarding substance use and head trauma. While an association is present, causal statements cannot be made from the results of the work. Recall bias is possible with self-reported measures. Questions regarding head trauma were broad, intentionally for this pilot year, but do not provide information on the timeframe between traumatic head injury and substance use. Recently, the relationship between head trauma and substance use has been mediated by mood disorders, specifically depression and anxiety (14). The questionnaire did not include items specific to mood disorders as it was outside the scope of the main research project (35). Therefore, those variables could not be added to the statistical models. To improve upon the noted limitations of this work, researchers should utilize a longitudinal design in a sample of AI/Ans with known substance use activities to track diagnosed and self-reported traumatic head injuries across the study timeline. In addition, mood disorders could also be documented to determine possible mediation of the association. Finally, there is the potential for the Hawthorne effect, as the self-reported data, particularly regarding sensitive topics such as TBI history and substance use patterns, could lead to underreporting or overreporting based on perceived expectations.

## 5 Conclusion

In assessing the relationship between substance use and head trauma in Native Americans who inject methamphetamines, we found that results differed based on self-reported gender. For females, the history of drug use influenced self-reported head trauma. In males, hypertension was a key factor. Most of the current literature focuses on alcohol consumption (12), is conducted in animals (12) or within the athletic population (14), and has limited representation of Native Americans, making the present results novel.

## Data Availability

The datasets presented in this article are not readily available because the datasets presented in this article must be granted only after having received approval from the Fort Peck Tribal Institutional Review Board. Requests to access the datasets should be directed to PF, PFiremoon@fpcc.edu.
